# The Allometry of Prey Preferences

**DOI:** 10.1371/journal.pone.0025937

**Published:** 2011-10-05

**Authors:** Gregor Kalinkat, Björn Christian Rall, Olivera Vucic-Pestic, Ulrich Brose

**Affiliations:** 1 Department of Biology, Darmstadt University of Technology, Darmstadt, Germany; 2 J.F. Blumenbach Institute of Zoology and Anthropology, University of Göttingen, Göttingen, Germany; Phillip Island Nature Parks, Australia

## Abstract

The distribution of weak and strong non-linear feeding interactions (i.e., functional responses) across the links of complex food webs is critically important for their stability. While empirical advances have unravelled constraints on single-prey functional responses, their validity in the context of complex food webs where most predators have multiple prey remain uncertain. In this study, we present conceptual evidence for the invalidity of strictly density-dependent consumption as the null model in multi-prey experiments. Instead, we employ two-prey functional responses parameterised with allometric scaling relationships of the functional response parameters that were derived from a previous single-prey functional response study as novel null models. Our experiments included predators of different sizes from two taxonomical groups (wolf spiders and ground beetles) simultaneously preying on one small and one large prey species. We define compliance with the null model predictions (based on two independent single-prey functional responses) as passive preferences or passive switching, and deviations from the null model as active preferences or active switching. Our results indicate active and passive preferences for the larger prey by predators that are at least twice the size of the larger prey. Moreover, our approach revealed that active preferences increased significantly with the predator-prey body-mass ratio. Together with prior allometric scaling relationships of functional response parameters, this preference allometry may allow estimating the distribution of functional response parameters across the myriads of interactions in natural ecosystems.

## Introduction

Despite decades of ecological research on species interactions, the vast complexity of most natural communities still challenges our understanding of population and community stability [1], [2]. The myriads of predator-prey interactions in complex food webs contrast negative complexity-stability relationships in random interaction networks [3]. As a general null expectation, they suggest that complex natural food webs should be unstable unless they possess non-random structures. Interestingly, theoretical research has demonstrated how the distribution of weak and strong interactions across complex food webs determines the community-level stability [2], [4]–[7]. In particular, research on body-mass constraints on interaction strengths and adaptive foraging has provided major mechanistic insights in these patterns [8]–[17]. Empirically, however, progress has been hampered by the lack of approaches that can be generalized across the myriads of interactions in complex food webs. Allometric functional responses predicting consumption rates by predator and prey body masses [18]–[22] and environmental temperature [23], [24] provide a critically important first step towards such generality. However, they focus on single-prey interactions while ignoring the complexity of natural communities, where predators are exposed to multiple prey. Here, we present an approach to generalize allometric interaction strengths from single-prey to multi-prey experiments.

**Figure 1:**
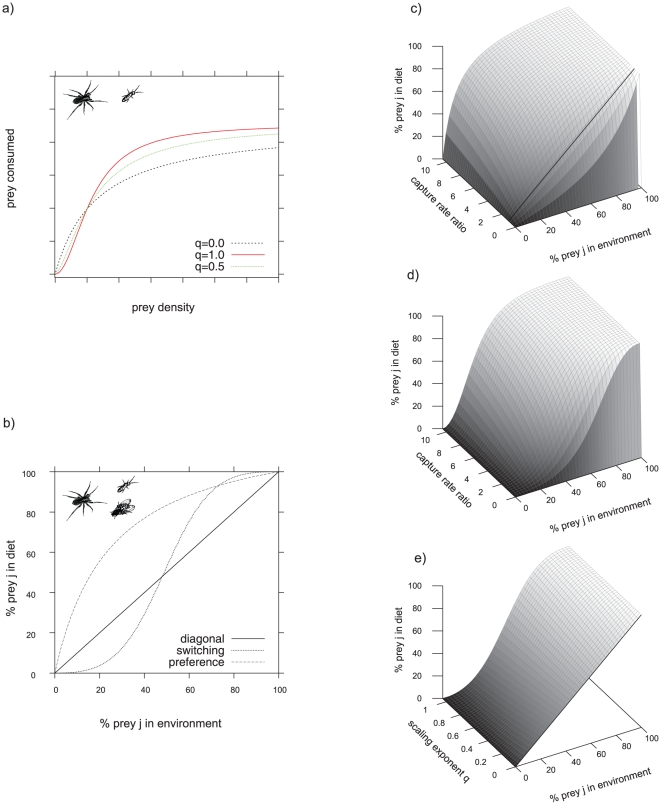
Conceptual illustrations of (a) type II and type III (single-prey) functional responses and the implications of variance in the scaling exponent *q* as well as consequences for *absolute* prey consumption and (b–e) preferences and switching in two-prey (here: *j* and *k*) experiments: b) “Traditional” preference plot with *relative* consumption depending on relative density of prey *j*: Consumption is strictly density-dependent (the diagonal solid line), or exhibits preferences for prey *j* (upper, long-dashed line) or switching behaviour (sigmoid, dotted line). c–e) Novel null model based on two-prey functional responses (Equation 3) with varying capture rate ratios (*b_ij_*/*b_ik_* with 0.01<*b_ij_*<10 and *b_ik_* = 1) for the two prey in c) type II (*q_ij_* = *q_ik_* = 0) and d) type III functional responses (*q_ij_* = *q_ik_* = 1). e) Gradual conversion of type II to type III functional responses when both prey are consumed with the same capture rate (*b_ij_* = *b_ik_* = 1). Constant handling time is used in figures c–e (*T_hij_* = *T_hik_* = 0.1). Note that the diagonal of strictly density-dependent consumption as the traditional null model (panel b) only emerges if both prey are consumed with exactly the same type II functional response (solid black lines in figures c and e).

One of the standard measures of interaction strength in food webs [25] is provided by predator-prey functional responses [26], [27] describing the per capita consumption rate of a predator, *F*, depending on prey density:
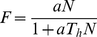
(1)where *N* is prey abundance, *T_h_* is the handling time needed to kill, ingest and digest an individual of the prey and *a* is the attack rate (hereafter: “capture rate” sensu [28]). This type II functional response with a constant capture rate can be modified to account for capture rates that vary with prey density, *a* = *bN^q^* [15], [29], [30], which yields type III functional responses:
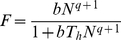
(2)where *b* is a capture coefficient (sometimes also referred to as search coefficient), and *q* is a scaling exponent that converts hyperbolic type II (*q* = 0) into sigmoid type III (*q>0*) functional responses (see Fig. 1a; note that some authors refer to intermediate or modified type II functional responses for values 0<*q*<1; e.g., [30]). The Hill exponent, *h*, used in some prior studies (e.g., [29]) is equivalent to *q* (*h* = *q*+1). Interestingly, the plethora of functional response studies concentrate on single-predator – single-prey studies (see refs [31]–[33] for an overview). Nevertheless, the question remains if these findings hold when predator and prey are embedded in the complex network of a natural community, where most predators have multiple prey.

To overcome this deficit we increased the complexity of the experimental setting by the comparisons of single-prey functional responses from a prior study [18] with two-prey experiments under identical experimental conditions, an experimental design rarely found in the literature (but see refs [34]–[36] for examples). Traditionally, however, most two-prey experiments that were designed as to investigate preference and switching behaviour have simplified this approach by (1) skipping the single-prey functional response experiments, and (2) varying the relative densities of both prey while keeping a constant total prey density [37]–[40]. These approaches are illustrated in Figure 1. The diagonal representing strictly density dependent consumption has often been used as the null model (Fig. 1b, solid line), and deviations from it were interpreted as preference for one prey (Fig. 1b, dashed line) or prey switching (Fig. 1b, dotted line) as an indicator of adaptive foraging behaviour [37]–[39], [41], [42]. Historically, the quest for switching and adaptive foraging behaviour has been fuelled by its stabilizing effect on population dynamics [15], [30], [43], [44]. One crucially important question remaining is whether strict density dependence (i.e., the diagonal in Fig. 1b) is a reasonable null model and consistent with predictions of the two single-prey functional responses. The functional response concept can be extended to a two-prey version:
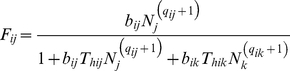
(3)where the per capita consumption of predator *i* on prey *j* depends also on the interaction between predator *i* and prey *k* [28], [44], [45]. Inserting the parameters of the two single-prey functional responses (*i-j* and *i-k*) in this model yields predictions of relative consumption within a two-prey experiment (Fig. 1c–e). If both single-prey functional responses are type II (i.e. *q_ij_* = *q_ik_* = 0), variance in the capture rates *b_ij_* and *b_ik_* (while *T_hij_* = *T_hik_*) can result in substantial variation in the predicted relative feeding rates of the two-prey experiment (Fig. 1c). Strictly density dependent consumption (i.e. the highlighted diagonal solid line in Fig. 1c) only emerges if *b_ij_* and *b_ik_* are identical. If both single-prey functional responses are of type III, sigmoid feeding curves are predicted for all combinations of capture rates, and the diagonal indicating density-dependent consumption does not occur on the predicted consumption plane (Fig. 1d). Thus, even if the two single-prey functional responses are characterised by the same handling and capture parameters (i.e., *b_ij_* = *b_ik_* and *T_hij_* = *T_hik_*) strictly density dependent consumption in the two-prey experiment is only predicted for pure type II functional responses (*q_ij_* = *q_ik_* = 0, Fig. 1e).

Together, these conceptual patterns have shown that strictly density-dependent consumption (i.e., the diagonal line in Fig. 1b) can only be used as the null model in two-prey experiments in the unlikely situation that both prey are consumed with exactly the same type II functional response. In all other cases, deviation from strictly density-dependent consumption can simply be a consequence of inherent characteristics of the predator-prey relationship (e.g. physiological or morphological constraints like limitations of the digestive system or gape-size limitation) manifested in different capture rates (and/or handling times as well as scaling exponents). Thus, the separation of active switching (i.e. switching behaviour deviating from single-prey based predictions) from passive switching (i.e. switching behaviour complying with single-prey based predictions) has been proposed [46]. We propose to further expand this concept by also separating active preferences (i.e. preference or avoidance behaviour deviating from single-prey based predictions) from passive preferences (i.e. preference or avoidance behaviour complying with single-prey based predictions).

Ecology has profited tremendously from replacing linear with non-linear null models in biodiversity research (i.e., neutral theory [47] or mid-domain models of biodiversity [48]). In the same vein, we propose that the wide-spread linear null model of strictly density dependent consumption is lacking realism and should be replaced by non-linear multi-prey functional responses. At the cost of increased complexity, they introduce more ecological plausibility and provide a deeper mechanistic understanding of predator-prey interactions. Subsequently, we will illustrate the use and potential of these non-linear null models in consumption experiments with terrestrial predators.

In the tradition of metabolic scaling models [49], [50], several studies dealing with a wide range of organisms revealed how capture rates (sometimes referred to as capture coefficients e.g., [51]) and handling time depend on body masses. In these relationships, handling times increase with increasing prey mass but decrease with increasing predator mass [18], [52]–[55], while capture rates follow hump-shaped relationships with predator-prey body-mass ratios [18]–[21], [52], [54]. Regarding the allometry of the scaling factor *q* we are not aware of any other study but the one by Vucic-Pestic and colleagues [18].

Here, we used allometric single-prey functional response models from a previous study [18] to predict the per capita feeding rates in two-prey experiments (Eqn. 3) using parameters from Vucic-Pestic and colleagues [18] to predict our two-prey experiments (see Methods section for details). We hypothesised that allometric functional response parameters should predict the consumption rates in the two-prey experiments thus resulting in “passive preferences” or “passive switching”. Alternatively, we aimed at explaining deviations from the multi-prey functional responses, equivalent to “active preferences” or “active switching”, by predator-prey body mass ratios.

## Materials and Methods

### Allometric single-prey functional responses

**Figure 2:**
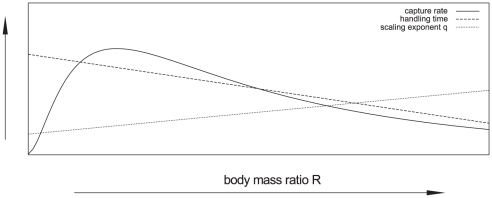
Conceptual graphic showing allometric relationships in the single-prey functional response parameters capture rate, handling time and the scaling exponent *q* as revealed by the previous study of Vucic-Pestic and colleagues [**18**].

**Figure 3:**
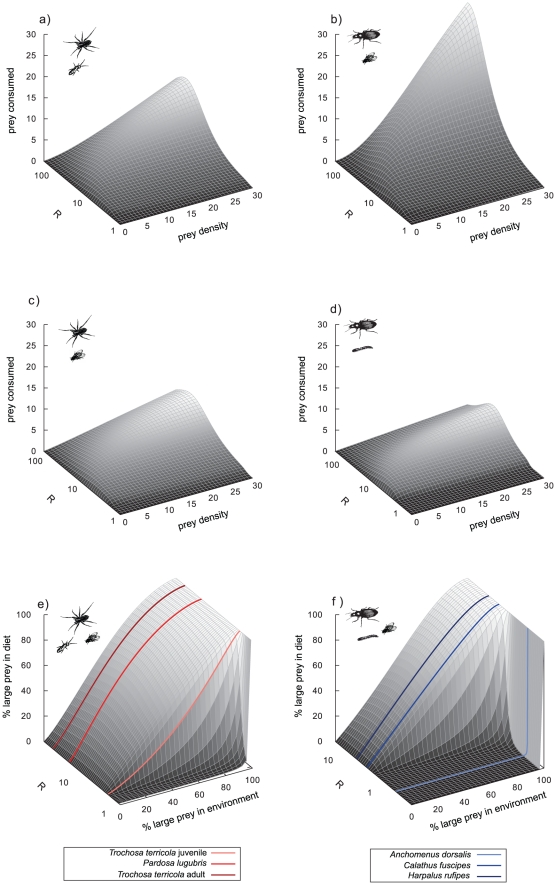
Single prey functional responses as a function of predator-prey body mass ratios from previous study[**18**] for the following predator-prey combinations: a) wolf spiders – *Drosophila*, b) ground beetles – *Alphitobius*, c) wolf – spiders – *Heteromurus* and, d) ground beetles – *Drosophila*. Parameters applied for these models are given in Table 1. Combining of the single-prey functional responses for one large and one small prey allowed calculating predictions of the allometric functional response models for the two-prey preference experiment with e) spiders (body-mass range from 1 to 200 mg) with *Drosophila* as large prey and *Heteromurus* as small prey, and f) beetles (body-mass range from 1 to 600 mg) with *Drosophila* as small and *Alphitobius* larvae as large prey. The coloured lines indicate the six species (i.e., body size classes) that were tested empirically in this study (see Fig. 4). Note the difference between *absolute* consumption in plots a–d while 3 e and f show *relative* consumption on the x- and z-axes. Note that for the two-prey plots (3 e and f) the predator-prey body-mass ratio (*R*) on the y-axes relates to the ratio between the predator and its larger prey.

In their study, Vucic-Pestic and colleagues [18] addressed systematic effects of predator and prey body masses on the functional response parameters handling time, *T_h_*, capture rate, *a*, and the scaling exponent *q* in experiments with 13 predator species comprising ground beetles and wolf spiders. The allometric dependence of handling time was estimated as:
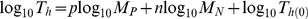
(4)with *M_P_* as predator mass, *M_N_* as prey mass, and *p*, *n*, *T_h(0)_* as constants. Furthermore, a hump-shaped relation for the capture coefficient *b* was defined as:
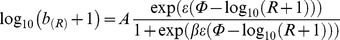
(5)where *A* is a constant, *Φ* represents the body mass ratio at which 50% of the maximum capture coefficient is reached, *ε* is the rate of change in search with mass controlling the steepness of the curve, *R* is the body-mass ratio (*M_P_*/*M_N_*) and *β* determines the asymmetry of the curve [18]. While handling time decreased with predator mass and increased with prey mass, capture rates followed hump-shaped relationships with predator–prey body-mass ratios ([18], Table 1, Figure 2). The scaling exponent, *q*, was low for predator-prey pairs with low body mass ratios (i.e. spiders - springtails and beetles – fruit flies) and high for the ones with high body mass ratios (i.e. spiders – fruit flies and beetles – lesser mealworm larvae) ([18], Table 1, Figure 2). These parameter combinations yield hump-shaped functional responses as presented in Figure 3 a–d.

**Table 1:** Parameters of the allometric two-prey functional response model as the null model for the preference experiment (Figs. 3 and 4): N = number of replicates; *M_P_* = average predator mass [mg]; *R* = average predator-prey body-mass ratio; *q* = scaling exponent; * parameters taken from ref [18].

	N	*M_P_*	*R*_(predator:large prey)_	*q*_(large prey)_*	*q*_(small prey)_*
**spiders**	207			0.17	0.52
*Trochosa terricola* juvenile	69	2.766	1.95		
*Pardosa lugubris*	70	28.895	20.35		
*Trochosa terricolsa* adult	68	78.874	55.55		
**beetles**	145			0.02	0.89
*Anchomenus dorsalis*	48	12.108	0.52		
*Calathus fuscipes*	49	65.712	2.83		
*Harpalus rufipes*	48	120.561	5.18		
Parameters applied in eqns. (4) and (5)°		*P* = −0.94; *n* = 0.83; *Th*_(0)_ = 0.35; *A* = 3.69; *ε* = 0.48; *Φ* = 0.45; *β* = 47.13;			

### Preference experiments

The experimental setting of our study followed the methods of previous studies [18], [21]–[24]: The predator individuals were kept separate in plastic jars dispersed with water and were deprived of food for at least 48 hours before the start of the experiments. The experiments were performed in Perspex® arenas (20×20×10 cm) covered with lids. The lids contained gauze covered holes to allow for gas exchange. The arena floor was covered with moist plaster of Paris (200 g dry weight) to provide constant moisture during the experiments. Habitat structure in the arenas was provided by moss (*Polytrichum formosum*, 2.35 g dry weight) that was first dried for several days at 40°C to exclude other animals and then re–moisturised prior to the experiments. Prey individuals were placed in the arenas half an hour in advance of the predators to allow them to adjust to the arenas. The experiment was run for 24 hours with a day/night rhythm of 12/12 h dark/light and temperature of 15°C in temperature cabinets. Initial and final prey densities were used to calculate the number of prey eaten. Control experiments without predators showed that prey mortality or escape was negligible.

The predator species represent a subset of those deployed within the previous study on allometric functional responses [18] including three wolf spiders (Aranea: Lycosidae) and three ground beetles (Coleoptera: Carabidae) that were weighted individually before the experiments. Consistent with predator body masses from the previous study [18], they were spanning a relatively wide range of body masses (Table 1). All animals in the experiments were either sampled by pitfall trapping outside protected areas around Darmstadt, Germany, or they were reared in laboratory cultures. Pitfall trapping was conducted at agricultural field sites with acknowledgment of land owners. None of the animal species involved are threatened of extinction nor is any one of them under protection.

Due to logistic constraints it was impossible within the present study to test the two-species allometric functional response model with all of the predator-prey combinations that were analysed in the previous study [18]. Nevertheless we used the same prey species and prey sizes of Vucic-Pestic and colleagues [18]: in the experiments with spiders the springtails *Heteromurus nitidus* (0.15 mg) and flightless fruit flies *Drosophila hydei* (1.42 mg) were deployed as small and large prey, respectively (hereafter *Heteromurus* and *Drosophila*). Meanwhile in the experiments with ground beetles the flightless *Drosophila* was the small prey while larvae of the lesser mealworm *Alphitobius diaperinus* (23.26 mg) were available as large prey (hereafter: *Alphitobius*).

Following the procedures of prior studies [22], [24] the overall prey densities in the preference experiments were kept constant at 30 individuals while systematically varying the relative prey densities between one small and 29 large prey individuals and 29 small and one large prey individual. Due to logistic constraints the experiments were carried out with ten and eight levels of relative density for wolf spiders and ground beetles, respectively. Each density level was replicated between six and eight times resulting in a total number of 352 experimental units. In the unique case of the ground beetle *Anchomenus dorsalis* (predicted capture rate on large prey *Alphitobius b* = 0) 58 replicates were discarded before statistical analyses because total consumption in these replicates was zero and thus calculating relative consumption was impossible. After the experimental duration of 24 hours, individual predators were removed and weighted and remaining individual prey were counted. Individual prey that were killed and partly consumed were counted as consumed. Individual predator weights before and after the experiments were then used to calculate individual average body weight.

### Models and statistical analyses

Figure 3 shows the single-prey functional response curves (from [18]) of the twelve predator-prey combinations that we tested within the present study: Fig. 3a shows the results for wolf spiders preying on the large prey species *Drosophila* and Fig. 3b shows the results for the small prey species *Heteromurus*. Fig. 3d and 3e show the single-prey functional response curves for three ground beetles preying on *Alphitobius* and *Drosophila* as large and small prey, respectively. The curves are plotted within a three-dimensional plot with body mass ratio *R* as *y*-axis to visualise the realised range of predator-prey body mass ratios. Additionally, we present the planes of the single-prey allometric functional response models that were derived from the previous study and subsequently applied to parameterise the two-prey functional response model predictions (see Table 1 for parameter values from [18]). The results of the model predictions for the two prey allometric functional response model are shown in figures 3c and 3f for wolf spiders and ground beetles, respectively, where the body mass ratio *R* on the y-axis represents the ratio between predator and the larger prey.

Note that both, the single-prey as well as the two-prey functional response model, assume a constant prey density throughout the experiment and the prey depletion following consumption was corrected by integrating over time and prey density [56], [57] (see [18] for more details). While single-prey functional responses allow analytical solutions, referred to as Roger's random predator equation [56], experiments with two prey required numerical integration. Therefore we inserted Equations (4) and (5) into Equation (3) and integrated the resulting equation over time (d*N_i_*/d*t* = −*F_ijk_*) to predict how feeding rates should behave in a two-prey predation experiment using the additional **R** package “deSolve” applying a Runge-Kutta 4^th^ order integration algorithm in **R** 2.11.1 [58], [59]. Equations (4) and (5) were parameterised according to empirical predator masses as well as parameter values from [18] (see Table 1) and two separate simulation settings were established for spiders and beetles, respectively (according to the different scaling exponents). Consistent with the experiments, the overall prey density (i.e., individuals of large prey plus individuals of small prey per arena) in the numerical simulations was set to 30 individuals while the experimental duration of 24 hours was split into 240 time steps (i.e., one time step = 6 minutes). The empirical results from the two-prey experiments were then compared to the numerically simulated prediction and checked for significant deviations by student's t-tests. Non-significant residuals (i.e., deviation of consumption from simulated two-prey functional response prediction) were interpreted as support for our initial hypothesis that allometric functional responses predict the consumption rates in two-prey experiments. Subsequently, we analysed the residuals by an ANCOVA using **R** 2.11.1 [58] to distinguish between effects of (1) the body-mass ratio between the predator and the large prey, (2) predator group (beetle or spider) and (3) level of relative initial density of the large prey.

## Results

### Numerical simulations of preference predictions

The results of the numerical simulations for expected passive preference patterns depending on predator body masses are shown in Figs. 3e and 3f for spiders and beetles, respectively. Despite differences in both, the scaling exponents *q* and the prey masses, the transition from predicted passive avoidance to passive preference for the larger prey occurs at a “tipping point” with body mass ratios of roughly two (i.e., predator is twice as large as the larger prey) for spiders and beetles. This phenomenon was recorded in both plots by an abrupt shift from zero consumption along all relative prey densities to strong preference for the larger prey within a relatively short range along the body-mass ratio axis. Interestingly, we did not find any indication of predicted passive switching (Figs. 3e and f).

### Two-prey experiments

**Figure 4:**
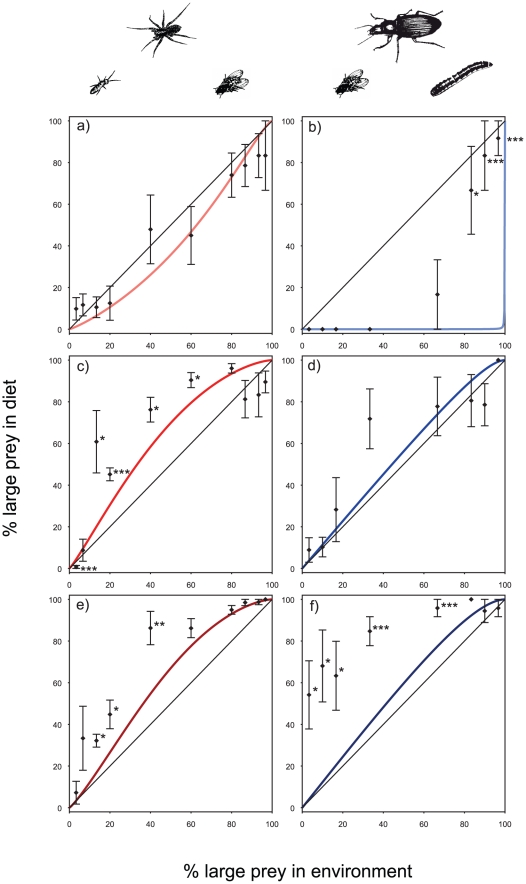
Two-prey consumption experiments for (a,c,e) spiders with *Drosophila* as large prey and *Heteromurus* as small prey, and (b,d,f) beetles with *Drosophila* as small and *Alphitobius* larvae as large prey. Solid black line indicates traditional null model of strictly density-dependent consumption, coloured lines show predictions of the allometric two-prey functional response model (see Fig. 3). Black diamonds show mean consumption in two-prey experiments, vertical bars indicate standard errors. T-test significance levels are indicated as: *<0.05, **<0.01 and ***<0.001. Panels show the results for a) *Trochosa terricola* juvenile, b) *Anchomenus dorsalis*, c) *Pardosa lugubris*, d) *Calathus fucscipes*, e) *Trochosa terricola* adult and f) *Harpalus rufipes*.

We tested the predictions of the allometric two-prey functional model for six predators: three spiders (predicted orange preference lines in Fig. 3e) and three beetles (predicted blue preference lines in Fig. 3f). The two-prey functional response models predicted passive preferences for the smaller prey in the experiments with the smallest spider (red line in Fig. 4a) and the smallest beetle (blue line in Fig. 4b), whereas all larger predators were expected to exhibit passive preference for the larger prey (Fig. 4c–f). Interestingly, the novel null model based on allometric two-prey functional responses (coloured lines in Fig. 4) differs in all experiments from the traditional null model of strictly density-dependent consumption (diagonals in Fig. 4).

Subsequently, we compared the empirical consumption rates to the two-prey functional response null model (coloured lines in Fig. 4). In four of the six two-prey experiments, we found substantial and significant deviations of the empirical consumption rates from model predictions (Fig. 4). This indicates active preferences for the larger prey by the wolf spiders *P. lugubris* (Fig. 4 c) and *T. terricola* (adult) (Fig. 4e) and the ground beetles *A. dorsalis* (Fig. 4b) and *H. rufipes* (Fig. 4f). Interestingly, the two predators that fulfil the criteria for passive preference are those with body mass ratios close to the “tipping point” of roughly two (*T. terricola* juvenile, Fig. 4a and *C. fuscipes*, Fig. 4d). The active preferences for the larger prey are evenly distributed across relative prey densities for *P. lugubris*, *T. terricola* and *H. rufipes* (Fig. 4c, e, f), whereas the smallest beetle, *A. dorsalis*, exhibited active preference for the larger prey only at the highest initial relative density of the larger prey (relative initial density >80%, Fig. 4b).

**Figure 5:**
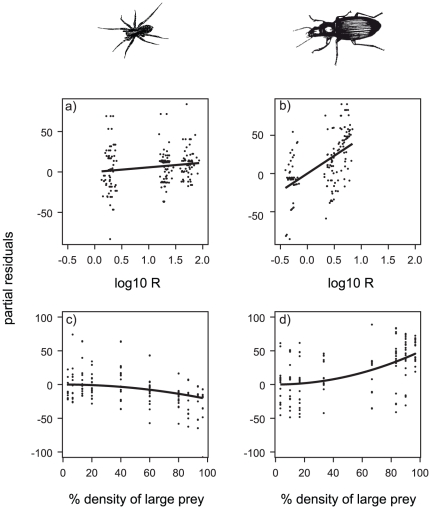
Active preferences (partial residuals) for the larger prey of (a, c) spiders and (b, d) beetles depending on the body-mass ratio between the predator and the larger prey (a, b) and the square of relative initial densities (c, d). Parameters: a) slope = 5.674, (s.e. ±2.594) intercept = 7.699 (s.e. ±4.734); b) slope = −0.002 (s.e. ±0.0004) intercept = 7.699 (s.e. ±4.734); c) slope = 46.575 (s.e. ±8.644), intercept = 5.227 (s.e. ±4.402); d) slope = 0.005 (s.e. ±0.0008) intercept = 5.227 (s.e. ±4.402).

A full factorial ANCOVA of the residuals revealed a highly significant three-way interaction term between predator group, square of relative initial prey density and the predator-prey body mass ratio (F_7,344_ = 26.41, p<0.001, r^2^ = 0.35). For subsequent more detailed ANCOVAs addressing this three-way interaction term, we separated the data sets into two predator groups. The ANCOVA of the beetles revealed a highly significant two-way interaction term between the square of relative initial prey density and predator-prey body mass ratio (F_3,141_ = 33.22, p<0.001, r^2^ = 0.41). In the spider data set, we removed the interaction term and found that predator-prey body mass ratio as well as the initial densities were significantly affecting the results (F_2,204_ = 16.76, p<0.001, r^2^ = 0.14). Interestingly, the residuals increased with predator-prey body-mass ratios for both predator groups (Figs. 5a and b), though the slope was much steeper for beetles (slope = 46.58±8.64 (s.e.), Fig. 5b) than for spiders (slope = 5.67±2.59 (s.e.), Fig. 5a). However, spiders and beetles responded differently in their active preferences to the relative initial density of the large prey: while spiders showed a weak negative relationship (slope = −0.002±0.0004 (s.e.), Fig. 5c), the relationship for the beetles was positive (slope = 0.005±0.0008 (s.e.), Fig. 5d).

## Discussion

In this study, we addressed the question whether laboratory functional response experiments combining predators with single prey species can predict the outcome of experiments with two prey species. Conceptually, we demonstrated that strictly density dependent consumption only emerges from multi-prey functional responses as the null expectation if both prey are consumed with exactly the same type II functional response. Employing empirical allometric two-prey functional-response models as a novel null model in our study yielded consumption rates that varied substantially from strict density dependence without implying any active foraging choices by the predators. We refer to these deviations as passive preferences. While the general pattern of passive preferences for larger and smaller prey with predator-prey body-mass ratios higher and lower than two, respectively, was correctly predicted by the two-prey functional responses, the majority of the predators exhibited additional active preferences for the larger prey. This consistent deviation from the null model suggests a general allometry of preferences.

### Simple null model

We illustrated the consequences of the popular fallacy of using strictly density-dependent consumption as the null model in two-prey experiments on preferences or switching behaviour. While some studies have correctly employed multi-prey functional responses as the null model (e.g., [34]–[36]), most prior studies avoided the labour-intensive development of all single-prey functional responses and used strictly density-dependent consumption as a more simple null model (e.g., [37]–[39], [41]). Our conceptual examples (Fig. 1 c–e) illustrate that this simple null model is only acceptable if both prey are consumed with exactly the same type-II functional response. As functional response parameters vary dramatically across different prey species (e.g., [21], [23], [33], [60]–[64]), we suggest that the simple null model of strictly density-dependent consumption will rarely apply. Unfortunately, this violation of the underlying assumptions invalidates the conclusions on preferences or switching drawn by many prior studies (e.g., [37]–[40], [42]).

### Allometric null model

We demonstrated how multi-prey functional-responses parameterised by single-prey experiments can be used as an alternative more adequate null model in two-prey experiments (Fig. 3 e and f). To avoid the labour-intensive study of all single-prey functional responses, we have proposed allometric functional response models as an alternative. These models represent systematic relationships between functional response parameters such as handling time and capture rate [18], [21]. The body masses of the species in two-prey experiments can parameterise these relationships that are subsequently entered in two-prey functional responses. Together, allometric relationships and two-prey functional responses provide novel null models predicting expected predator consumption rates if the co-occurrence of the two prey does not influence the interactions. Certainly, allometric scaling relationships might provide inaccurate estimates of functional-response parameters. In this study, the twelve individual single-prey functional responses necessary to parameterise the six two-prey models were available from a prior study [18]. However, predictions based on these single-prey functional responses were entirely consistent with those of the allometric functional response models. We have thus decided to base the presentation of the null model in the present study on the allometric functional-response models, because they will allow a more wide-spread application in other studies where the single-prey functional responses are not necessarily available. In our study, the steep rise from zero consumption for low body mass ratios (here: ratio between predator and large prey: *R*≤2) regardless of the relative prey densities is consistent for both predator groups and may be due to the steep rise in attack rates with body mass ratio on the left hand side of this hump. One has to bear in mind that this well documented hump-shaped relationship arises from different constraints on foraging rates at the two different sides of the hump [13].

In this study, variation in the body-mass ratio was only included at the level of the individual predators that were weighted for every single treatment. At the prey level, however, we worked with fixed average sizes for the three prey species resulting in fixed body-mass ratios between large and small prey for all treatments, because data on prey of other sizes were not available from the previous study [18]. Therefore, future studies on allometric functional responses (*i.e.*, single-prey *and* multi-prey studies) should include more variation in prey body size to extend the allometric functional response concept. Nevertheless, the allometric concept provides a general framework for parameterising interaction strengths within complex food webs.

### Passive and active preferences

Moreover, the allometric two-prey functional responses take inherent characteristics of predator-prey relationships into account and thus allow deeper mechanistic understanding of predator choices. Most importantly, the novel null model allows to clearly separate between passive and active preferences. We define “passive preference” as a deviation from strictly density-dependent consumption driven by morphological, physiological and behavioural (evolutionary) adaptations that constitute a specific predator-prey interaction in both the simplified (i.e., one prey) as well as a more complex (i.e., multiple prey) environment. In contrast, “active preference” implies significant differences among simplified and more complex environments induced by short-term behavioural changes (e.g., different rate of attacks upon encounter if an alternative prey is present). Our analyses show passive as well as active preferences, and they allow separating the body-mass constraints leading to passive preferences from predator choices yielding systematic active preferences for the larger prey by most predators. We refer to this entirely novel and systematic pattern as the “allometry of preferences”.

### Passive and active switching

Interestingly, our systematic exploration of the novel null model demonstrated the potential for passive switching if the passive preferences of the predator switch between prey depending on their relative density. This phenomenon is generally expected if both single-prey functional responses are of type III. Although the spiders in our experiments exhibited a type III functional response on both prey, the consumption rates predicted for the two-prey experiments did not include any passive switching.This apparent contradiction is explained by the numerical integration procedure to account for prey depletion during the experiments: The low prey densities in the numerical simulation of the two-prey model prevented passive switching. However, incorrectly using the simple multi-prey functional response without accounting for prey depletion yielded predictions of slight passive switching among prey. Generally, we would expect passive switching only if both single-prey functional responses were more strongly sigmoid (closer to a “true” type III functional response with *q* = 1) and thus both scaling exponents were considerably higher than 0.2 [30].

To our knowledge, laboratory studies that found switching predators mostly introduced this effect by the design of the study through providing distinct sub-habitats (e.g., [35], [41]). While the predators in these studies were “forced” to change their foraging mode according to the distribution of the different prey items, our study was designed to provide a uniform habitat. Nevertheless, the exploration of our two-prey null model suggests that type III functional responses can cause passive switching, which is counter-intuitive compared to conventional wisdom in population ecology [28], [46]. While strongly stabilising effects of adaptive foraging in theoretical studies [65] have triggered a quest for empirical documentation of switching (e.g., [42]), we caution that adaptive foraging requires active variation in prey preferences, which cannot be deduced from sigmoid consumption rates crossing the diagonal line of strictly density-dependent consumption. Our results stress the need to adopt more sophisticated null models such as the allometrically parameterised two-prey functional responses to provide empirical support for adaptive foraging.

### Experiment

In our experiments, four of the six predators showed active preferences for their larger prey indicated by significant deviations from the null model predictions. Meanwhile we found passive preferences close to a density dependent consumption for the small spiders (juvenile *T. terricola*) as well as for the intermediately sized beetle (*C. fuscipes*). Interestingly, all predator-prey interactions with body-mass ratios larger than two (adult *T. terricola*, *P. lugubris* and *H. rufipes*) exhibited strong active preferences for the larger prey. This entirely novel preference allometry is supported by statistically significant increases in active preferences with predator-prey body-mass ratios. Moreover, the relative densities of the two prey species exhibited additional effects on preferences, which were skewed in opposite directions for beetles and spiders. Despite this opposite effect of relative prey densities and differences in the strength of the increase in preference with body-mass ratios between beetles and spiders, this general pattern allows more accurate generalisations of functional responses across the myriads of interactions in complex food webs. Our analyses may also explain a more general pattern that larger carnivorous mammals focus on large prey, whereas small carnivorous mammals focus on small prey as revealed in a large meta-study [66].

### Caveats

We found a different pattern of active preferences for the larger prey by the smallest beetle *A. dorsalis*: despite a low body-mass ratio (0.52) we found active preferences for the larger prey at the highest relative prey densities. While a previous single-prey functional response experiment indicated that these large prey are too big to overwhelm and ingest for the small beetle *A. dorsalis*, we found in the present experiment that single events of this feeding interaction occasionally occurred (though the results are somehow skewed as we had to discard 58 replicates for zero consumption). One possible explanation is that we could not control the body masses of every prey individual, and *A. dorsalis* overwhelmed particularly those prey individuals smaller than the average mass of 23 mg. Additionally, *A. dorsalis* was not able to ingest the whole prey individual in the experimental time of 24 hours. Moreover, *A. dorsalis* is relatively inefficient at catching the smaller prey, flightless *Drosophila*: For instance, the capture rate of similarly sized spiders on *Drosophila* is two orders of magnitude higher (*b* = 36.3 for *A. dorsalis* compared to *b* = 1500 for *P. lugubris*) [18]. Together, these specific constraints on *A. dorsalis* may explain how stochastic effects have caused active preferences at the highest relative prey densities. In principle, however, this example highlights that allometric models trade predictive power in specific cases for the sake of gaining generality across species. Interestingly, the concept of allometric functional responses is flexible to incorporate phylogenetic constraints [21] which allows tailoring accurate models for specific experiments.

### Conclusions

Our conceptual approach demonstrated that the wide-spread use of the simple null model of strictly density-dependent consumption is impeding mechanistic advances. Instead, progress requires application of more sophisticated null models for two-prey experiments such as the allometric two-prey functional response. Our analyses revealed systematic patterns of active and passive preferences. In particular, the majority of predators actively preferred the large prey. If this finding of a systematic preference allometry generalises across additional predator groups and other ecosystem types, we anticipate that it may provide towards a general understanding of constraints on interaction strengths in natural communities. This may have substantial importance in creating the patterns of many weak and few strong interactions that stabilise natural food webs.
